# Neonatal care and community-level treatment seeking for possible severe bacterial infection (PSBI) in Amhara, Ethiopia

**DOI:** 10.1186/s12913-020-05081-0

**Published:** 2020-03-30

**Authors:** Meron D. Asfaha, Dawn L. Comeau, Sydney A. Spangler, Brandon L. Spratt, Lamesgin Alamineh, Abebe G. Gobezayehu, John N. Cranmer

**Affiliations:** 1 Emory University, Atlanta, GA USA; 2 Emory University – Ethiopia, Bahir Dar, Amhara Ethiopia; 3 Emory University – Ethiopia, Addis Ababa, Ethiopia

**Keywords:** Ethiopia, Possible severe bacterial infection (PSBI), Sepsis, Neonatal mortality, Care seeking, Community-based care

## Abstract

**Background:**

In Ethiopia, neonatal mortality accounts for approximately 54% of under-five deaths with the majority of these deaths driven by infections. Possible Severe Bacterial Infection (PSBI) in neonates is a syndromic diagnosis that non-clinical health care providers use to identify and treat newborns with signs of sepsis. In low- and middle–income countries, referral to a hospital may not be feasible due to transportation, distance or finances. Growing evidence suggests health extension workers (HEWs) can identify and manage PSBI at the community level when referral to a hospital is not possible. However, community-based PSBI care strategies have not been widely scaled-up. This study aims to understand general determinants of household-level care as well as household care seeking and decision-making strategies for neonatal PSBI symptoms.

**Methods:**

We conducted eleven focus group discussions (FGDs) to explore illness recognition and care seeking intentions from four rural kebeles in Amhara, Ethiopia. FGDs were conducted among mothers, fathers and households with recruitment stratified among households that have had a newborn with at least one symptom of PSBI (Symptomatic Group), and households that have had a newborn regardless of the child’s health status (Community Group). Data were thematically analyzed using MAXQDA software.

**Results:**

Mothers were described as primary caretakers of the newborn and were often appreciated for making decisions for treatment, even when the father was not present. Type of care accessed was often dependent on conceptualization of the illness as simple or complex. When symptoms were not relieved with clinical care, or treatments at facilities were perceived as ineffective, alternative methods were sought. Most participants identified the health center as a reliable facility. While designed to be the first point of access for primary care, health posts were not mentioned as locations where families seek clinical treatment.

**Conclusions:**

This study describes socio-contextual drivers for PSBI treatment at the community level. Future programming should consider the role community members have in planning interventions to increase demand for neonatal care at primary facilities. Encouragement of health post utilization could further allow for heightened accessibility-acceptability of a simplified PSBI regimen.

## Background

In 2017, 15,000 children died per day contributing to about 5.4 million under-five deaths globally [1]. Roughly 54% of all under-five deaths are driven by deaths in the neonatal period [2]. Although notable progress has been made in reducing under-five mortality, neonatal mortality rates have decreased at a much slower rate; from 1990 to 2017, under-five deaths have undergone a 58% reduction compared to 49% of neonatal deaths [1]. The majority of neonatal deaths (75%) are caused by prematurity, intrapartum related events and neonatal sepsis with sepsis alone contributing to an estimated one out of four neonatal deaths [3]. In Ethiopia, neonatal mortality remains a public health concern; approximately 95,000 neonates died in Ethiopia in 2017 [1]. Additionally, the 2019 Ethiopian Mini Demographic Health Survey (EMDHS) reflects a neonatal mortality rate of 30 deaths per 1000 live births during the 5 years preceding the survey [2]. Nationally, the burden of absolute and proportionate neonatal mortality remains elevated. In rural, agrarian regions the persistent mortality burden is largely driven by limited availability and accessibility of clinical services due to geographic distance between households and facilities or financial costs of transportation [4–7]. Regionally, neonates in Amhara, Benishangul-Gumuz and Tigray have a higher risk of mortality compared to their peers in Addis Ababa [8]. However, there remains limited region-specific data on the precise determinants of neonatal death in Amhara.

Within communities and primary care clinics, neonatal infection is usually diagnosed based upon a set of signs and symptoms that lead the clinician to suspect sepsis. This syndromic diagnosis of sepsis is defined as Possible Severe Bacterial Infection (PSBI). Clinical criteria of PSBI are algorithmically determined per The Young Infants Clinical Signs Study Group [9] and include fast breathing (≥ 60 breaths per minute), severe chest-in drawing, fever (≥ 38 °C), hypothermia (< 35.5 °C), no movement or movement upon stimulation only, poor or no feeding and convulsions [10]. First-line facility-based treatment for PSBI consists of a 7-day course of injectable antibiotics (either procaine penicillin plus gentamicin or ampicillin plus gentamicin) [10]. However, accessing facility-level care is not always feasible, possible or desirable, particularly in rural low- and middle-income country contexts. Further, obtaining financing for sick newborn care may lead to catastrophic financial consequences [11]. For instance, low-income families in Enugu, Nigeria averaged care expenditures totaling 157% of their monthly family income for neonatal care [12]; such large financial implications are not uncommon in LMICs. In agrarian settings, peripheral health facilities such as health posts in Ethiopia [13], are more readily accessible to communities than hospitals. This is often due to limited transportation, low household financial resources, or varied sociocultural factors that limit the accessibility or desirability of hospital-based care. When curative PSBI treatment at the hospital is not possible, Health Extension Workers (HEWs) have the ability to identify and treat PSBI symptoms using a simplified antibiotic treatment plan [14]. Despite global guidance on PSBI care outside of inpatient health facilities, PSBI treatment coverage at rural health posts and within communities remains low.

Across Ethiopia, documented determinants to accessing biomedical care for newborns at health centers or hospitals include distance to health facility, cost of transportation, and social care seeking norms [13]. At the primary level, the decentralized Ethiopian Health Tier system includes a primary hospital, health centers and rural health posts [13]. Although HEWs may refer those seen during home visits or at health posts to higher facilities for care, significant barriers limit community members in accepting these referrals. Sociocultural factors preventing care at health facilities include differences in gender-based priorities and decision-making dynamics at the household level which may delay seeking care for sick neonates [15–17]. Barriers impeding demand of care for newborn treatments include fear of newborn exposure to environmental factors (i.e. sunlight), newborn isolation from strangers until the newborn has been religiously blessed and ambiguous newborn personhood [7, 14, 17]. However, only three known studies assess the determinants of care for sick newborns in Amhara [6, 18, 19] with no known studies assessing household care seeking for neonatal PSBI in the region. Although findings are specific to the woredas sampled in this study, recommendations may be transferable by local and regional contexts. This study aims to understand household care seeking and decision-making strategies for neonatal PSBI symptoms. Due to limited availability of data on drivers of newborn and infant care in Amhara, investigators also sought to identify general determinants of care at the household level.

## Methods

### Study setting

Two *woredas* (districts) within a 300-km radius of Bahir Dar (capital city of Amhara) were selected for inclusion in this study. Both woredas comprise a majority of rural residents and were selected based on data from the 2007 Population and Housing Census of Ethiopia, Statistical Report for the Amhara region. Indicators such as population size, number of households and type of settlement (urban versus rural) were considered during site selection (Table 1). Number of households varied between woredas. Woreda A comprised 6405 households with 767 households in Woreda B. Four rural *kebeles* were purposively selected with the goal of representing various influencers for PSBI decision makers in rural communities.

**Table 1:** Characteristics of *woreda* sites

Zone	Woreda and ***kebele***	Number of households^a^	Population size	Rural	Urban
West Gojam	Woreda A	6405	292,080	269,403	22,677
	*Site 1*	*1348*	*5764*	–	–
	*Site 2*	*7719*	*1832*	–	–
East Gojam	Woreda B	767	132,883	130,299	2584
	*Site 3*	*2332*	*10,183*	–	–
	*Site 4*	*1640*	*8082*	–	–

Source: Central Statistical Agency – Ethiopia. *The 2007 Population and Housing Census of Ethiopia: Statistical Report for Amhara Region.* Addis Ababa, Ethiopia; 2012

^a^Households refer to housing units, per the Central Statistical Agency – Ethiopia definition

### Study design

Qualitative research methods were employed to identify care seeking determinants for neonatal possible severe bacterial infection (PSBI) in rural Amhara. The methodology for this study was informed by existing studies analyzing determinants of health-seeking behavior (such as illness perception and characterization) in low- and middle-income contexts [20]. Data were collected through focus group discussions (FGDs). The discussion guide was initially prepared in English and translated into Amharic. FGDs were led by one moderator and one note taker, with observations documented by the principal investigator to document setting, behavior and contextual information for analysis. All discussions were conducted in the Amharic language and interviews were backtranslated into English. FGDs were stratified amongst mothers, fathers and household units. The purpose of collecting information from FGDs with mothers, fathers and household units was to understand household responsibilities and community-level decision making for neonates with signs of PSBI.

Mothers in rural Ethiopia are typically primary caretakers of the child, although approval from fathers is often needed to follow-through on decisions requiring financial resources. Additionally, fathers are typically regarded as household decision-makers. For this reason, participant groups were separated by parental roles. Focus groups of household units, including all members of the household concurrently, were included to understand responses that may be influenced by power dynamics i.e. how maternal responses were given in the presence of the father. Household units typically comprised mothers, fathers and peripheral family members such as grandmothers and siblings of the newborn.

### Recruitment

Prior to accessing *kebele* sites, the research team (one moderator, one notetaker and the investigator) met with health center directors, health center supervisors and health extension workers (HEWs) at their respective locations to describe the study’s purpose. HEWs were approached and functioned as a recruiting mechanism, sampling participants from the catchment area of health centers. HEWs were asked to purposively identify households with newborns from health center records, utilizing the defined inclusion criteria, and targeted eight to ten participants per kebele. Discussions with the selected participants were scheduled over the phone or through approaching households by foot. Participants were selected if they had a newborn in the household within the previous 2 years, were 18 years of age or older and had newborns with PSBI symptoms (symptomatic group, SG) or were residents of the target communities (community group, CG). CG data was provided to compare care methods and trajectories among participants that were not recruited based upon a pre-specified ailment or condition.

Participants identified in the SG were chosen if they had a newborn in the household exhibiting one or more PSBI symptoms in the first 28 days of life, as documented in health center records. PSBI symptoms were defined per the 2015 WHO guidelines and included fast breathing, chest in-drawing, fever, hypothermia, no movement or movement only upon stimulation, poor feeding or no feeding, and/or convulsions [10]. Community group (CG) participants were purposively selected regardless of the newborn’s health status based on their residence in the target communities. HEWs selected participants that were accessible or approachable via foot or vehicle for recruitment. The sampling frame included ten discussions per woreda (Table 2). Final recruitment consisted of 11 FGDs due to participant unavailability in some woredas.

**Table 2:** Sampling strategy, per *woreda*

Target Group and Collection Method	Number by woreda^a^	Total Recruitment Goal^a^	Total Recruited
Mothers (FGD)	2 (5–8)	4 (10–16)	4 (29)
Fathers (FGD)	2 (5–8)	4 (10–16)	4 (13)
Household Members (FGDs)	2 (5–8)	4 (10–16)	3 (9)
Total	6 (15–24)	12 (30–48)	11 (51)

* Numbers are reported per the number of groups and range of participants in each group

### Data collection

Data were collected between July and August 2018. The moderator and note-taker were both experienced in qualitative data collection. The moderator, fluent in Amharic and English, was additionally trained as a clinical nurse. FGDs typically lasted 45–75 min. Focus group guides included questions on household decision-making, care-taking actions and responsibilities, illness causation and characterization, illness severity, decision-making power and methods or facilities for care (Additional files 1, 2 and 3). Although introduction questions specifically addressed the newborn period, some questions were generalized to extend responses towards infancy.

Discussions with mothers and fathers were conducted at health post compounds. Focus groups with household members (including the mother, father and peripheral family members such as brothers, sisters or grandparents of the newborn) were conducted in participant households. Demographic information was collected after discussions to gather information on age, number of people residing in the household, income, occupation, and education level. For mothers, information on the number of live births and number of children was collected to determine child loss.

**Fig. 1:**
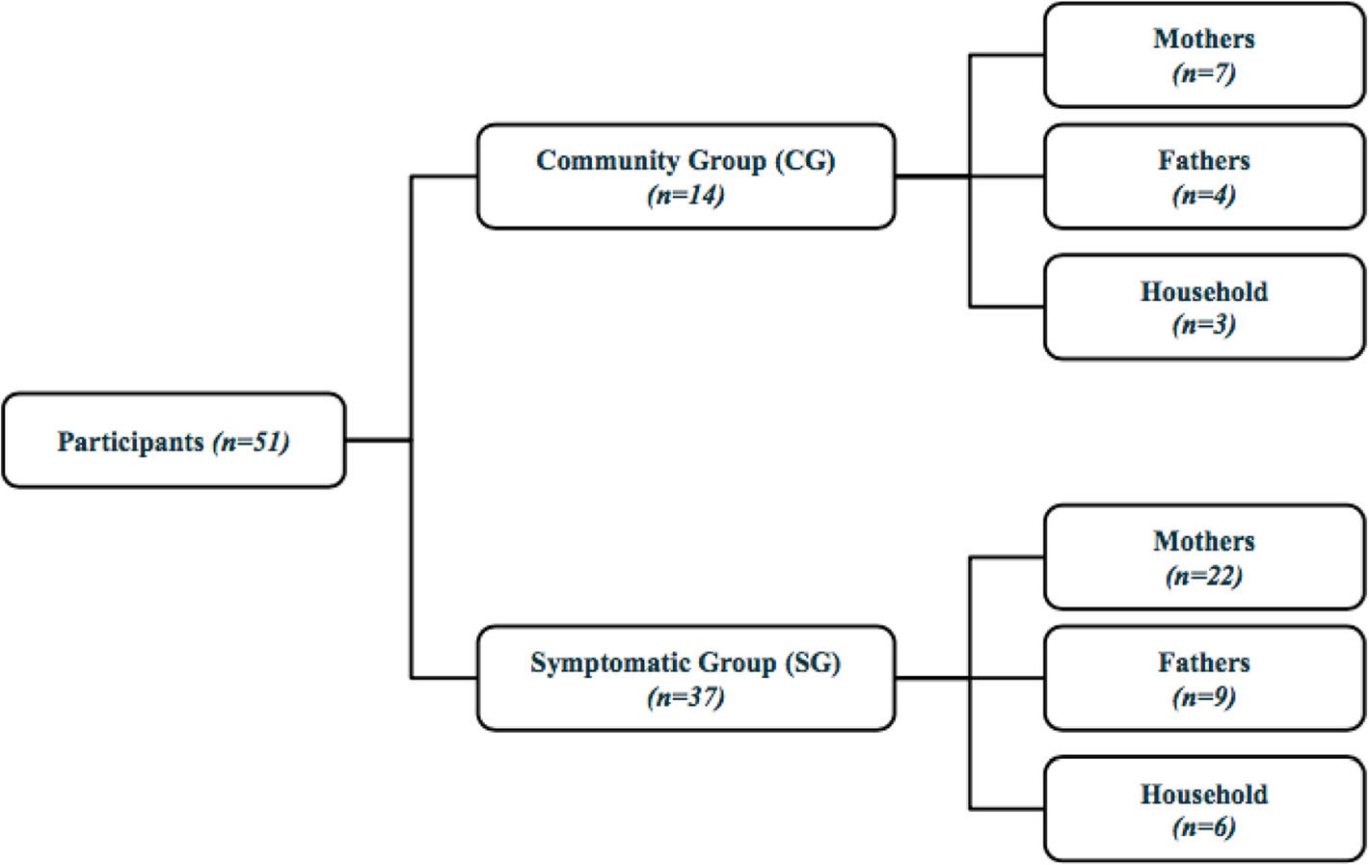
Recruitment outcome

Precision of qualitative instruments were improved after reviewing preliminary data from the first two focus groups. Using pre-testing and in-field revision, the research team identified opportunities to utilize additional probes to improve richness of data. The research team additionally decided to focus on recruiting SG participants to ensure understanding of PSBI-related illness recognition, characterization and care seeking behaviors. As a result, after three CG focus groups, data collection focused on the SG to ensure saturation on the experiences of SG mothers, fathers and households (Fig. 1).

As a result of this recruitment strategy (Table 2), 14 participants met CG recruitment criteria and 42 participants met SG recruitment criteria (Fig. 1). Four FGDs with mothers (29 participants), four FGDs with fathers (13 participants) and three FGDs with households (9 participants) were across the West and East Gojam zones. Of all FGDs, five were conducted in *Woreda A* and six in *Woreda B*.

All discussions were recorded utilizing audio recorders. After data collection ended, data were transcribed into English by one bilingual Amharic-English transcriber with ample qualitative research and transcription experience. To assure quality of translation, three transcripts were re-transcribed by the same transcriber to affirm data quality. Discussions amongst the study team were continued throughout analysis to increase clarity when statements were made within the local context of communities.

### Analysis

Data were thematically analyzed utilizing MAXQDA software (version 18.1.1, Berlin, GA, 2018). Thematic analysis is an iterative analytical approach to identifying concepts (themes) in the transcriptions [21]. Analytic memos were first created to contextualize emerging patterns and concepts. Memos largely addressed three questions: 1) How do different family members play a role in newborn care and care decisions? 2) Where did participants go to seek treatment for the newborn? and 3) How were these methods or facilities accessed? A codebook was then developed inductively using concepts that emerged while reading transcripts.

Three transcripts were independently coded by the principal investigator and one research assistant experienced in qualitative research. During discussions of each transcript, agreement was sought to increase intercoder reliability. A final codebook was then created using input from two investigators. Emerging themes were further identified through iterative analytic memos, transcript summaries as well as analysis of key code intersections. Key codes were extrapolated then compared across sampling methods, sites and types of family members. Frameworks were created and revised throughout analysis to conceptualize study findings. Coding, memos and interim analyses were then discussed across the team—including the lead investigator, senior authors, faculty and data collectors.

### Ethics

During the consent process, participants were informed of the study’s purpose, procedure and implications. Verbal consent was obtained as most respondents did not read or write in Amharic. The Emory University Institutional Review Board (IRB) determined data were exempt from full human subjects research review (July 3, 2018). Activities were approved by the Amhara Public Health Institute (APHI) ethical review board (July 18, 2018). Letters of support were provided to West and East Gojam zonal health departments prior to data collection.

## Results

### Participant characteristics

Fifty-one participants took part in FGDs (Table 3). Thirty-seven respondents had symptomatic newborns (SG) while 14 were community members (CG). Rural kebeles were selected in both woredas; 21 respondents came from Woreda A and 30 respondents came from Woreda B. There were 29 respondents in focus groups with mothers, 13 in focus groups with fathers and nine participants in household focus groups.

**Table 3:** Demographic Characteristics of All Participants (SG and CG)

		% (N)	Median (Range)
**Number of Family Members**			4 (3–10)
**Age (years)**			28 (18–54)
**Last Year of Formal Schooling**	No Formal Education	49% (25)	
	Primary Education	37% (19)	
	Secondary Education	12% (6)	
	Degree or higher (bachelors and/or post-graduate)	2% (1)	
**Occupation**	Merchant/Petty trade	43% (22)	
	Agriculture	43% (21)	
	Skilled Labor	14% (7)	
	Unskilled Labor	14% (7)	
	Housewife	25% (13)	
	Other: Student	3.9% (2)	
	Professional/Technical/Managerial	2% (1)	
**Ethnicity**	Amhara	100% (51)	
**Religion**	Christian (Orthodox)	100% (51)	
**Annual Household Income**			14,400 birr (495 USD)
**Number of Live Births**			2 (1–8)
**Number of Living Children**			2 (1–8)
**TOTAL (*N*** **= 51)**			

(a) Number of family members, Age (years), Annual Household Income, Number of Live Births and Number of Living Children reported in median and range values. Occupation responses were not mutually exclusive, some participants reported having more than one occupation. (b) Median (Range) is an absolute range

All participants identified as being of Christian Orthodox faith and were of Amhara ethnicity. The median respondent age was 28 years and median household size was 4. Participants primarily reported working in agriculture or as merchants (66.7%) with a median annual household income of 14,400 birr (approximately 495 USD). There were substantive variations in obstetric history with 31.3% of all mothers experiencing a child loss and mothers having a range of 1 to 8 live births.

### Key themes

Themes from the data included family decision-making methods for newborn care. We summarized community-level experiences related to newborn care, relationship-based roles within the household, illness identification strategies and preferred treatment approaches based on illness type. Consequently, five key themes emerged from these data: maternal responsibility for the newborn; maternal decision-making; environmental, hygiene and nutrition practices as drivers of illness; illness conceptualization based on complexity; and care seeking trajectory.

#### Maternal responsibility for the newborn

Mothers generally reported having primary responsibility for providing general care to newborns. While participants from FGDs regarded both parents as instrumental for newborn and infant care, mothers were consistently identified as the primary caretakers across respondent groups—particularly in the first 2 weeks of life. Most participants indicated the mother’s key role is to breastfeed and/or provide breastmilk. Some participants indicated maternal breastfeeding was one of the few actions households could take to protect the newborn’s health during the first 2 weeks of life. Additionally, respondents indicated mothers were responsible for maintaining the child’s hygiene using practices such as washing the newborn’s clothes and cleaning the newborn’s body with baths. During the infant period, participants additionally mention protecting the child from being exposed to dirty objects or environments such as from contaminated water or placement of miscellaneous items into the mouth. Other maternal caretaking roles during the newborn period include preparation of the newborn’s sleeping space (preparing the “mat”), relaxing or playing with the child and protection from harm or diseases. Specific actions that were taken to protect the newborn included avoiding sunlight, safeguarding the newborn from cold weather and utilizing mosquito nets while sleeping.

Maternal responsibility for the newborn was often described with regards to the provision of nutritional nourishment and dietary care. This is described in a dialogue between fathers:

*F3: From my knowledge the mother should eat different types of balanced foods such as energy-giving foods. Up until 6 months the infant should feed from the mother’s breastmilk only.*

*I: What about others? As a father what do you do to make your child healthy?*

*F1: There is nothing more than this. It is the same as he explained. Before, we gave [food] for the infant to make his body grow faster but it became harmful so we don’t give other foods up until 6 months of age. Because of this until the child grows bigger we don’t do anything but the mother performs most of the care. The infant doesn’t take the other foods we feed to the mother.*

*(Fathers, PSBI, Woreda A)*

Participants mention the importance of adhering to a diet of breastmilk up until 6 months of age in order to maintain the child’s health. In assuring these dietary needs were met, participants remarked that there is “nothing more than the mother” as mothers are regarded as essential for newborn care provision. However, fathers and peripheral family members such as siblings and grandparents of the newborn indirectly cared for the child.

Paternal actions for newborn care primarily included securing materials such as soap and food for the child. Additional paternal actions included transporting the newborn to health facilities, namely the health center, for care during times of illness or to receive vaccinations. For instance, several fathers mentioned they assured medications were given to the child as prescribed by health workers, although information on the types of medications taken were not discussed. Actions taken comprised preparation of food and coffee for the mother while she was away at the health center or washing the child’s clothing for the parents.

After the newborn period, respondents identified various nutritional practices. For instance, participants in FGDs with mothers and FGDs with fathers self-identified that when the infant becomes 6 months of age, the child’s diet is expanded to include complementary foods. Food items provided after 6 months of age include cow milk, bread, eggs and roasted barley flour (*besso*). One participant described giving raw butter (*kibeh*) to the newborn, however this was only mentioned when referencing a traditional dietary practice that is now considered harmful and does not widely occur in the community. Prior to 6 months, participants repeatedly mentioned the necessity of breastmilk as a protective mechanism for newborn and infant health.

#### Maternal decision-making

Mothers were often identified as the primary decision-makers for newborn healthcare. This included seeking care for sick infants at health facilities or using informal, non-biomedical methods. Mothers reported they typically decided how and where the newborn received care for illness. However, this contrasts with what fathers reported. Fathers reported that both parental figures are generally responsible for making healthcare decisions. These respondents mention that actions taken for treatment are only made after discussions amongst both parents regarding the child’s state of health. In some cases, fathers also mentioned that the mothers are primary decision-makers for care, although this was reported less frequently than in focus groups with mothers. However, when mothers are unable to make care decisions due to their own ailments or not being present at the time of child illness, fathers indicate that their role was to do so themselves.

Beyond the primary caretaking role of mothers, fathers explicitly appreciated mothers taking initiative in the choices made on how to care for infants that were sick were commended. One father remarks:

*F: We simply accept her decision. For example, when she makes decisions about family situations, maybe for health conditions, we accept and help her to be effective in her decisions.*

*(Household, CG, Woreda B)*

Most members of the household, in fact, valued maternal decision-making. Types of care decisions that were appreciated were both preventative and curative in nature. These included securing vaccinations for the newborn or active follow-through on visiting formal or informal care services, particularly when the father was not present due to working “in the town” or in their farms. Fathers generally expressed agreeance to maternal decisions, at times explicitly expressing appreciation (as seen above). Some household members explained that this appreciation was due to the amount of time fathers usually spent outside of the house. As such, fathers were not always present when newborn illnesses were recognized and next steps for care were deliberated.

#### Environmental, hygiene and nutrition practices as drivers of illness

Participants across groups and locations reported newborn illnesses were primarily caused by gaps in hygiene practices or unmet maternal nutritional needs. Two hygiene-drivers of newborn illness were identified. One included an unclean home environment while the other considered exposure of the newborn to polluted water which could introduce bacteria and cause illness. Maternal nutrition drivers included improper dietary habits such as high consumption of alcohol or having an imbalanced diet, such as ingestion of diverse foods that may not be agreeable to the mother. Improper foods also comprised those that would not contribute to the mother’s vitality and were not “energy-giving.”

One mother lists causes of newborn illnesses and states:

*M: [It occurs] when we feed them contaminated foods, when we do not keep the children clean, exposing the newborn baby with cold air and when we do not put them to sleep properly.*

*(Mothers, PSBI, Woreda B)*

External environmental and spiritual causes of illness were less commonly identified. Exposure to cold weather and wind were generally identified as factors contributing to illness. Further, specific sleeping habits, such as sleeping without a mosquito net or without properly preparing the sleeping mat, was identified as a cause. Finally, evil eye could also cause infant illness although respondents indicated protective measures such as visits to traditional healers and herbal remedies could avert the effects of evil eye for infants.

Most participants reported that severe consequences could result if a family fails to access the health center when an infant is ill. Without biomedical care at health centers or hospitals, participants expressed that some infants could die or suffer further illness progression. However, in the case of severe adverse outcomes such as death, participants saw this as a spiritually-driven outcome. In some cases, participants referred to serious consequences as fate or “God’s will.” For example, one mother who recently lost a daughter comments on her child’s outcome:

*I: Maybe if your child got better medical treatment do you believe she may have been cured?*

*M3: No, I do not think so. It was fatal. She couldn’t reach the next referral. God didn’t allow her to grow up and be mine.*

*(Mothers, PSBI, Woreda A)*

Although maternal responsibility in nurturing the child was emphasized, blame for illness or death was not placed on any household member. Instead, unsuitable outcomes were either reported as being caused by environmental and social factors (nutrition, hygiene and evil eye) or lack of preventative care. Limited treatment services at the health center were mentioned as contributors of illness exacerbation. For example, one father noted the lack of supplies (medication) at the health center that are suitable for various types of illnesses. Another father stated that going to the health center could “waste time,” particularly when the child is sick and needs immediate attention. However, the aforementioned factors that may potentially intensify illness progression were not frequently mentioned.

#### Illness conceptualization based on complexity

Approaches to identifying child illness were similar among household respondents (mothers, fathers, family members) and between households across regions. Families primarily identified symptoms of concern such as fever (high body temperature), abnormal breathing, lack of feeding or no feeding. Families also mentioned non-PSBI symptoms including abnormal or frequent crying, vomiting and diarrhea. Participants often attributed the onset of symptoms or illness to the lack of precautionary hygienic measures, such as keeping the child away from dirty environments or bathing regularly; lack of regular feeding patterns; and poor weather leading to cold temperatures and wind.

Illness conceptualization thus led to various methods of care according to the treatment that was deemed appropriate for the particular illness. Some participants described their child’s ailments as either “simple” or “complex.” These descriptions were based on the perceived severity of the illness and in some cases subsequently defined how families made decisions for treatment, due to how the illness was characterized. “Simple” cases were referred to when symptoms, or the onset of symptoms, were easily understood whereas “complex” cases were indicated when symptoms, or their onset, could not be explained. Simple and complex cases both included crying, behavioral changes (i.e. irritability) and physiological (i.e. temperature) changes. However, complex symptoms regarded as indicative of serious disease were abnormal breathing and lack of response, as mentioned by one father. What differentiated simple or complex characterization was how rapidly symptoms developed as well as how symptoms were understood. In complex cases, participants sometimes remarked that the disease was or might have been too severe for biomedical treatment. For simple, “common” or “ordinary” cases, participants mention seeking care at health centers. One father comments:

*“When the disease is simple we take the child to the health center but when it is complicated we will take the child to the church for holy water treatment.”*

*(Fathers, PSBI, Woreda A)*

These characterizations of illness informed decisions for where families sought care. Similar to this response, another father comments on the alternative methods used to treat “emergency” cases where the the illness involved a drastic or sudden onset of symptoms that could not be justified. He remarks:

*I: Do you use traditional medicine to treat child sickness?*

*F: Yes, we do.*

*I: What kinds of medicine do you use?*

*F: When there are sudden diseases that the health workers cannot understand, at that time we use traditional medicines. The children suddenly cry or become silent and their temperatures drop, at that time we use traditional medicine in emergency cases to help sick infants.*

*(Fathers, PSBI, Woreda A)*

Through this dialogue, the onset of signs and symptoms that were least understood consisted of the child being quiet, the child suddenly crying or a sudden decrease in temperature.. Although non-biomedical (traditional) medications were considered in these cases, participants did not often refer to them as their first choice for care. Further, receiving holy water at the Orthodox Church was viewed primarily as a preventative strategy to ward against future illness.

#### Care seeking trajectory

All participants mentioned healthcare facilities as a primary method for treating sick newborns. Specifically, health centers were referred to as the fundamental source of curative care for childhood illnesses. Health posts were not reported by any participants as a location to seek care for sick newborns. However, participants reported visiting health posts to receive preventative care, such as newborn vaccinations, when needed. When noticing that the newborn was sick, only one participant stated that they did not access the health center when the newborn was sick.

If families first sought biomedical treatment for sick infants that was deemed ineffective, most families then sought alternative care. These options included either holy water (*kurban*) or traditional medications. Holy water was received at churches as a strategy to avoid ailments for children and parents. Medicinal herbal treatments were obtained through traditional healers and used to prevent or cure evil eye. In addition, traditional therapies and healers were the primary resource for treating complex illnesses or used when the illness cause was not well understood. This care seeking pathway is described by one father during a CG focus group in Woreda B:

*F: Most of the time the community goes to the health center when the child becomes sick. After the health center, if not cured, we go to other places to treat our sick child. First we use medical treatment and then holy water treatment...if they’re not cured we use traditional medicine... if they’re not cured we come back to the health center and get medical treatment but we complain about why they don’t refer us to the hospital because there we have medical insurance. After that, the community makes their own choices. Either they go for holy water treatment or they use traditional medicine.*

*(Fathers, CG, Woreda B)*

The health center was the facility that participants most often went to when recognizing that the child was ill. Multiple participants mentioned that due to their health insurance coverage, there was no reason not to visit health centers. During a household discussion, one father comments:

*“At this time everyone goes to the health center. We pay one time a year for health treatment so, we go to the health center when there are any problems. At this time there is health insurance and we pay once a year, when there are any little problems we rush to the health center but before this program we had shortage of money. Due to this we used traditional medicines to cure our sicknesses but now we visit the health center. For those that have no money the government covers all medical costs. In our town there are more than 40 people that the government covers all expenses for medical treatment.”*

*(Household, SG, Woreda B)*

Although insurance status was mentioned on several occasions, only a few participants commented on their own universal healthcare status (whether they were covered with an insurance plan or not).

**Fig. 2:**
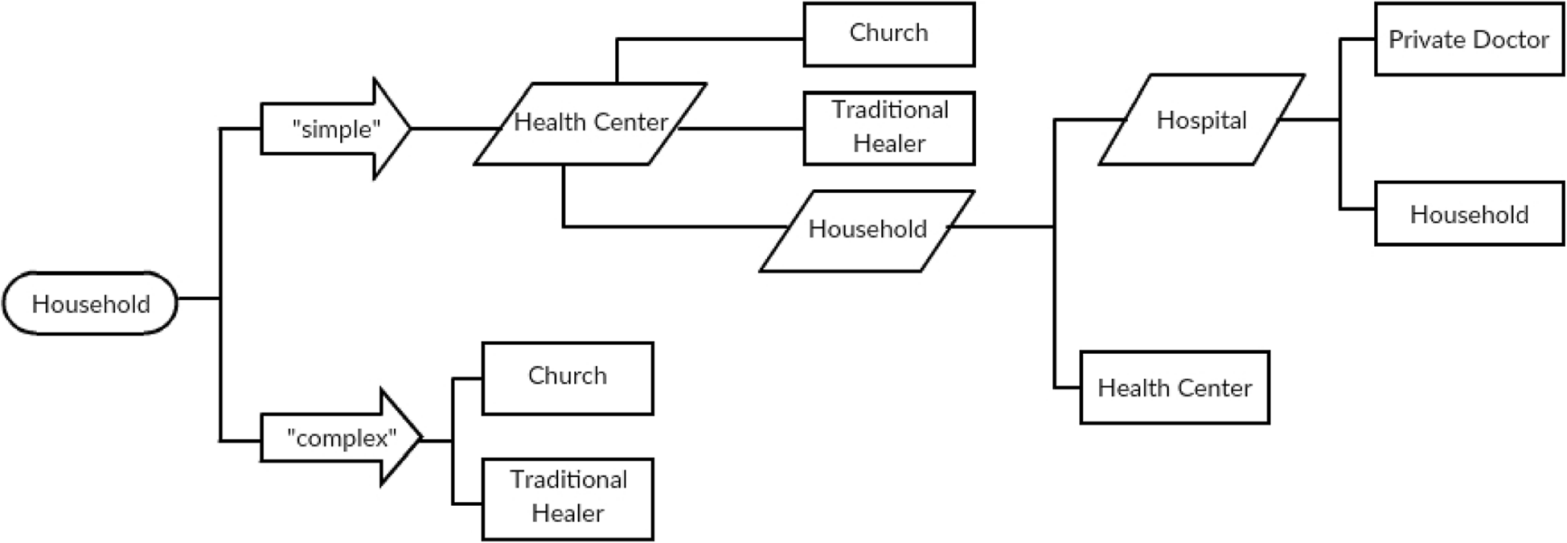
Care trajectory

When illnesses were unresolved, alternative methods such as home treatments and the hospital were subsequently sought (Fig. 2). Failure to visit biomedical facilities for care was at times due to observed ineffectiveness of medical treatments. In one CG focus group, fathers mentioned forgoing a HEW’s advice to avoid the removal of tonsils in their sick children. This was due to repeated occurrences of child deaths from tonsillitis within the community. Upon noticing these child deaths and recurring infections in their own children, they opted to either visit the traditional doctor or self-perform removal of tonsils. However, forgoing medical advice was not common and never occurred without first visiting a biomedical facility. Participants repeatedly recommended the health center for treatment when recognizing symptoms indicating illness in the newborn.

## Discussion

Strategies for community-based, HEW-delivered care have been proven effective and feasible in settings where referral to a hospital is not possible. However, treatment demand at community-level health facilities remains low. The aims of this study are to describe household neonatal care and decision-making strategies for neonatal PSBI symptoms. Although there are several existing studies on treatment seeking for newborns with illnesses in low- and middle-income countries (LMICs) [6, 22–24], this study may be the first to assess the determinants of securing treatment within the community for newborns with PSBI. This qualitative study outlines five key themes for neonatal care and management for PSBI symptoms: maternal responsibility of the newborn; maternal decision-making; environmental, hygiene and nutrition practices as drivers of illness; illness conceptualization based on complexity; and care seeking trajectory. Findings generated from this study will be synthesized into an implementation science portfolio for a decentralized and simplified antibiotic regimen strategy.

Understanding the roles different household members have in providing neonatal care is critical to informing targeted public health programming. Across focus groups, mothers were repeatedly acknowledged as significant for maintaining or improving newborn health, a finding consistent with another study in the region [6]. This was often due to the nutrient demands (breastfeeding) and time required of mothers during the newborn period. This finding suggests that mothers have many opportunities to detect newborn illness. As such, public health programming such as HEW and HDA home visits may largely focus their efforts on mothers to influence demands for care at outpatient facilities. Health campaigns that involve community members in targeted health messaging may promote newborn care seeking as a strategy to increase the demand for services at the health post and to create a sense of the individual’s autonomy in care acquisition [25].

Although most family members in our study were encouraging of the mothers’ autonomy to make care decisions, fathers often mentioned reminding the mother of newborn care duties such as providing medication regimens for the child on time. Another study conducted across Nigeria, Tanzania and Ethiopia finds fathers act as authoritative figures of whom agreeance is needed to follow through with accessing treatment methods, even though these figures may not often directly involve themselves in newborn care [26]. As timing of care is an essential component of ameliorating health conditions and preventing neonatal death [17, 27], increasing paternal awareness of neonatal danger signs through acceptable campaign messaging may facilitate transition from illness recognition to facility access.

Across contexts, recognition of neonatal danger signs are often proposed as a determinant of the types of care methods sought as well as care timeliness [6, 23, 28]. Symptoms that indicated illness among parental figures most often included fever, lack of breastfeeding and excessive or abnormal crying. All of these symptoms have been identified in a similar study on newborn care seeking practices in Central and Southern Ethiopia [29]. Several participants identified newborn illnesses as either “simple” or “complex.” Some who specifically characterized the newborn’s illness as complex described their newborn’s illness as being too severe for successful clinical care due to perceived ineffectiveness of biomedical treatment. Delays in newborn care seeking have also been reported when the condition of the child was perceived to be hopeless or too severe [7]. Unique to this study is that some families characterizing newborn illness as “simple” mentioned visiting the health center because there was no reason not to. Health insurance was often commented as justification for liberally visiting health centers if the child became ill (“there is no reason not to visit the health center”). Since 2010, Ethiopia has implemented and scaled up community-based and social health insurance in rural areas [30, 31]. This may explain why families in this study rarely mentioned finances as a barrier to accessing the health center.

Participants frequently and strongly indicated sick newborns should be cared for at health centers. Logistical barriers such as distance to facilities and time were rarely mentioned as obstacles in assuring biomedical treatment. However, many studies conducted in LMICs recognize geographic accessibility as a significant impediment to achieving care, particularly in rural settings [32–34]. This contrast may have been because study participants were recruited from the catchment area of local health centers and thus, participants may not have been likely to reside in hyper-rural areas far from health facilities. Most constraints on accessing care for sick newborns were thus related to how illnesses were conceptualized. This is a key finding in support of multisectoral partnerships for improved healthcare provisions and scale-up of the health insurance scheme in Ethiopia. Further, there are discrepancies between pre-service rural HEW education and the day-to day reality of rural HEW tasks [35]. To improve recognition of neonatal danger signs amongst community members, pre-service HEW education for rural HEWs could emphasize the importance of universal healthcare seeking regardless of illness conceptualization as “simple” or “complex” to further strengthen the link between household illness recognition and biomedical care acquisition.

Interestingly, the health post was not mentioned at all as a viable care facility for sick newborns. This may be an artifact of the HEW-based purposive sampling from health center records since participants were selected from the catchment area of health centers due to their accessibility during HEW recruitment. Health posts functioned as one solitary room within the health centers visited. Mother and father focus group discussions occurred in health posts nested within the local health center. Therefore, some participants may not have differentiated between services offered at health posts and health centers because of their shared locations.

Studies should aim to increase understanding of poor care seeking behaviors at health posts. Implementation strategies can enhance proven approaches in low- and middle-income country contexts. To do so, action should be taken to incorporate community mobilization, expand community health worker strategies (HEWs and HDAs) and enhance community-based interventions of which community members are involved in the development and implementation of programming efforts, to amplify acceptability of care seeking learned approaches [36].

### Limitations

Although this study aimed to sample households with symptomatic newborns (SG) and community members (CG), this sampling frame did not yield divergent responses to care seeking strategies for sick newborns between CG and SG households. Since many childhood illnesses share symptoms (fever, diarrhea, poor feeding), the similarity in findings across CG and SG groups may represent commonly shared approaches to childhood illness at the community level. Additionally, to obtain an adequate sample for analysis, we included any households that had a sick newborn in the previous 2 years. This extended timeframe may contribute to recall bias and diminish respondents’ specificity about PSBI-specific care seeking behaviors. Future studies may compare responses from families with sepsis-confirmed newborns versus community members to gain an additional understanding of PSBI/sepsis-specific care in this setting or limit the sample to respondents whose newborns were more recently ill—perhaps in the previous one to 2 months.

## Conclusions

This study contributes to knowledge on household-level newborn care and drivers of newborn treatment seeking in rural Amhara, Ethiopia. It suggests care seeking strategies and trajectories in this context may be widely shared at the community level. Consequently, strategies to engage community members in defining and creating decentralized approaches to PSBI care are indicated to maximize their relevance, accessibility and desirability. In the Ethiopian health system context, engaging peripheral health workers (HEWs) and volunteers (HDAs) may be a salient strategy for creating a decentralized model of care, particularly in understanding “simple” and “complex” symptoms as identified by community members and various subsequent care pathways. Community-based interventions, of which community members are involved in the development and implementation of programming efforts, may serve to strengthen the identification of these symptoms while amplifying acceptability of learned and normative care seeking behaviors regardless of simple or complex illness identification [36]. Encouraging home visits using HDA volunteers may be one powerful strategy for bringing PSBI care and treatment closer to the households—particularly in contexts where geographic or contextual barriers limit the uptake of life-saving PSBI care at referral facilities or hospitals. However, in instances where community members do not view or acknowledge the health post as a sufficient facility to care for newborns, emphasis on reinforcing the health post and health centers in HEW training may prove helpful.

## Supplementary information

**Additional file 1.** Focus group guide for recently-birthed women.

**Additional file 2.** Focus group guide for fathers.

**Additional file 3.** Focus group guide for households.

## Data Availability

The datasets generated and analyzed during the current study are not publicly available to protect confidentiality of participants in the communities studied. However, these data are available from the corresponding author upon reasonable request.

## Abbreviations

FGD: Focus Group Discussion
HEW: Health Extension Worker
HDA: Health Development Army Volunteer
PSBI: Possible Severe Bacterial Infection
