# Supplementary material for: Neonatal care and community-level treatment seeking for possible severe bacterial infection (PSBI) in Amhara, Ethiopia
Source: BMC Health Serv Res. 2020 Mar 30;20:264. doi: 10.1186/s12913-020-05081-0 (PMC7106804; doi:10.1186/s12913-020-05081-0)
Supplement: Supplementary file 1 — Additional file 1. Focus group guide for recently-birthed women. [file 12913_2020_5081_MOESM1_ESM.docx]

**Focus Group Guide**

**Recently-birthed women (FG#1)**

**Introduction**

Thank you for joining us today. I understand all of us have had a child within the past two years.

1. Please tell us the name of your child and what you did to care for them when they were newborns.
   - What were your responsibilities?
   - How do others in your community care for newborns just after childbirth? (first days and weeks)

**Newborn Care and Illness**

Let’s talk about newborn care and illness. Sometimes people get care for their babies to keep them healthy or when they are sick.

1. Tell me about what you do to keep your baby healthy - particularly when they are newborns.
   - What actions did you take to care for your baby? (i.e. church blessing, traditional healer, clinic appointments, immunizations)
   - Where did you go for care? (i.e. home only, church, health post, health centre, hospital)
   - How do others care for the newborn? (i.e. friends, family members, community leaders, health specialists)
2. Please describe a time when you thought your newborn was sick. How did you know they were sick? (i.e. community members, health specialists, symptoms)

- Fast breathing?
  - Hot to touch?
  - Cold to touch?
  - Chest draws in?
  - Moves only when stimulated/no movement?
  - Poor/stopped feeding?
  - Convulsions (seizures)?

1. How do you make decisions about getting care for your newborn?
   - Who makes decisions for newborn care in the household?
   - How did others influence your decision to seek care?
   - What are the reasons you did or did not go to a clinic? (health post, health centre, hospital)

**Newborn Infection and Treatments**

I would now like to discuss your thoughts about infections and treatments.

1. What actions did you take when your newborn was sick?
   - How did you treat your newborn? (traditional healers, home treatments, religious ceremonies, medications)
   - How effective were these measures in treating your newborn?
   - Where did you go to get care? (traditional healers, religious leaders, health post, health centre, hospital)
2. In your community, what measures are taken to care for a baby with an infection? (traditional treatments, home treatments, medications, religious ceremonies)
   - What resources are available?
3. Describe your experiences with newborn death in your community.
   - What do you think the causes of death are for sick newborns?
   - What happens to newborns that do not receive treatments when they are sick?

- What happens if newborns do not go to a clinic when they are sick? (health post, health centre, hospital)

1. Is there anything else related to newborn health, illness and infections in your community that you would like to discuss?

*Thank you for sharing. We hope that this discussion was informative. Your responses are valuable and provided a depth of information on the experiences shared within this community. Please remain seated for a couple of follow-up questions. If you would like any additional information on this discussion, please do not hesitate to let me know.*
